# Reticulated Round Cell-Sarcoma of the Orbit, with Secondary Growths Internally Containing Melanotic Deposits

**Published:** 1882-01

**Authors:** William Soula


### IV.—RETICULATED ROUND CELL-SARCOMA OF THE ORBIT, WITH SECONDARY GROWTHS INTERNALLY CONTAINING MELANOTIC DEPOSITS.

REPORTED BY WILLIAM SOULA.

Gray mare, æt. 12. There was first noticed a small tumor over the left eye-ball, apparently growing from the orbital wall, twelve months prior to death. During the first few months it grew very slowly.

In May, 1881, an incision was made into the tumor to determine positively whether it was solid or contained fluid, blood oozed from the puncture, but the mass was solid. During the next four weeks the growth gradually increased in size. Late in June it was again cut into and a small portion removed for microscopic examination. "The examination of this small portion showed that the growth was sarcomatous of the large round cell variety with a slight tendency to an alveolar structure. No melanotic spots were found as we so frequently meet with in tumors of the eye and in various parts of the body in white and gray horses."

"At this time a diagnosis of sarcoma was rendered, advising an early operation. The prognosis given, however, was that the animal would be improved by the operation, but that it would return at the original site or internally."

After this partial removal, for two months, the mare continued in fair condition, worked every day, but the growth continued to enlarge slowly.

In the early part of October she suffered from the disease known as "pink-eye," being unable to work for about ten days. By this time the growth had attained considerable size, was growing more and more rapidly, pushing the eye-ball forward, filling the orbit, and was causing the animal a good deal of pain.

She was, however, again put at her work, and accomplished the same with ordinary ease. From this time on it was noticed that she gradually lost flesh and strength, probably from the pain and annoyance of the orbital growth as well as from general disease.

October 23d, 1881, the animal was totally unfit for duty, and an operation was decided upon. The tumor, which was quite firmly attached to the orbital wall and completely filled the orbit, so that the eye-ball, in fact the whole contents of the orbital cavity, was removed by Dr. E. Benj. Ramsdell. The animal suffered severely from shock, but rallied completely at the end of a few hours. The wound was brought together by a few sutures, and cold dressings applied. The wound healed kindly with scarcely any suppuration. The animal improved so rapidly that two weeks after the operation she was again at work. For a few weeks she gained in flesh and strength.

Two weeks after she was at work, it was noticed that the breathing was hurried and impeded. This interference with respiration gradually increased until December 10th, 1881, when the animal was no longer able to work, and was placed in the hospital.

Two days later a physical examination was made, and revealed fluid in the left pleural cavity. A few days later a small quantity of fluid was detected in the right pleural sac. Four days before death the left chest cavity was aspirated, and one gallon of clear serous fluid drawn off. After the removal of the fluid marked and extensive consolidation of the left lung was diagnosticated, and thought to be due to secondary deposits, as none of the rational symptoms of pneumonia were present. Some consolidation was also detected on the right side. The left pleural cavity rapidly refilled with fluid, and the animal died four days later, or on December 21st, 1881.

"*Necropsy* a few hours after death. The wound of operation remained perfectly healed, and no evidence of return of the growth was present.

"*Thoracic cavity.*—The right pleural cavity contained about eight gallons of clear serous fluid; the left fully as much, if not more. The diaphragm was a thickened mass of new growths. The costal pleura was covered with rounded tumors, varying in size from that of a pea to that of a hen's egg. The right lung was thickly studded with pinkish white masses, varying much in size. Most of the growths, but especially the smaller ones, were of soft consistency; some of the larger masses seemed to contain fibrous elements as well. New growths were distributed through the left lung, over the left costal pleura and in the left diaphragm, but were more abundant than upon the right side.

"Some of the abdominal organs seemed to be involved, but owing to the incompleteness of the necropsy, nothing positive could be determined relating to the same.

"*Examination of the tumors*, by Prof. William H. Porter, at the School of Histology.—Macroscopically, the tumors were whitish in color, mottled with a pale pink, in some, dark (almost black), spots were present, as if they were deeply pigmented. The smaller masses were very soft when isolated and easily broke down under the slightest pressure. When only a single neoplasm existed at a given point, it seemed to be encapsulated by a thin layer of distinct tissue.

"*Microscopic examination.*—Scrapings of the juice from the cut surface showed only large round connective tissue and blood corpuscles. Sections from the tumors showed, at points, a distinct reticulated structure closely packed with these large round cells. At other points large round cells, imbedded in a homogeneous matrix, was all that could be seen. The growth resembled somewhat a medullary carcinoma. The growths, however, were very vascular and contained numerous extravasations of blood. The black points recognized by the naked eye, appeared under the microscope like the deposits found in melanotic sarcoma. It was, without doubt, blood pigment, and not masses of carbon particles, so commonly met with in the lung tissue. In sections made from the tumors found in the lung substance, numerous yellow elastic fibres were seen running through them, also, in some instances, the true pulmonary tissue. This condition would readily lead one to suppose that the new material had been deposited in and around the vesicular or lung tissue, and not primarily at its expense. In some of the large masses considerable fibrillated connective tissue had been developed, forming numerous bands running in various directions. This is one of the most malignant forms of sarcoma met with.

"The microscopic appearance of the primary tumor in the orbit and the secondary deposits were almost identical."

[This specimen is of special importance to the comparative pathologist and also to the veterinary practitioner. It is a third specimen of sarcoma which has been examined at the School of Histology in which the growth differed in each case, so that we have already met with three distinct varieties of sarcoma that are common to man. They also came from different kinds of animals, as the dog, cow and horse. In this connection it might be well to state that we have also met with a carcinomatous growths in the cow and horse. This, however, is not the first time we have seen a sarcoma in horses. This case, in connection with other cases reported in this number and previous issues, show that the various forms of malignant growths known to occur in man are probably quite as common in the lower animal, and consequently calls for a closer observation on the part of the veterinarian, thus giving increased impulse to comparative pathological study and the advancement of comparative medicine, for true advance depends upon a more thorough pathological knowledge of the diseases of the lower animals, and until then progress will be slow.

This case is of marked surgical importance, and brings up many interesting points which the surgeon cannot learn too quickly. The improvement, even after the late removal, can teach but one thing, which is that all tumors must be removed early if we wish to attain the best results possible. In dealing with malignant growths this rule should never be forgotten—*cut wide outside the growth*—for pathological examinations so often reveal the fact that only the major portion of the growth has been removed, thus leaving behind a nucleus, as it were, for a rapid recurrence. In this case you will notice that the whole orbital contents were removed, and the tumor did not return in the orbit. It seems quite just in this case to infer that the internal organs were invaded at the time of the operation.

In view of that, however, the operation was certainly justifiable; the only mistake was in not operating earlier. We think every one can clearly see that the mare's condition was temporarily improved and that she was relieved of great suffering, and the life and usefulness of the animal was prolonged.

Some might argue that if you remove a malignant growth it will return, either in the original site or internally. This, however, is not a good reason for not operating, for it has been found that repeated operations do prolong life, and in case it returns internally it is a great blessing, from the well-known fact in human medicine that these internal growths cause less suffering than the external. And it seems to me that it should be the grand purpose of every veterinary surgeon to devote his life to relieving the sufferings of lower animals in every way known to a rapidly advancing science.

The case is also of interest in a medical view, showing how accurately diseases of the chest cavity can be diagnosticated by the aid of a thorough knowledge of physical diagnosis. It also shows that the contents of the chest cavity can be drawn by the aid of an aspirator, with very little, if any, danger of the fluid becoming purulent as a result of the operation.—Ed].

